# Biomechanical influence of lateral meniscal allograft transplantation on knee joint mechanics during the gait cycle

**DOI:** 10.1186/s13018-019-1347-y

**Published:** 2019-09-05

**Authors:** Yong-Gon Koh, Jin-Ah Lee, Yong-Sang Kim, Kyoung-Tak Kang

**Affiliations:** 1 Joint Reconstruction Center, Department of Orthopaedic Surgery, Yonsei Sarang Hospital, 10 Hyoryeong-ro, Seocho-gu, Seoul, 06698 Republic of Korea; 2 Department of Mechanical Engineering, Yonsei University, 50 Yonsei-ro, Seodaemun-gu, Seoul, 03722 Republic of Korea

**Keywords:** Finite element analysis, Gait cycle, Meniscal allograft transplantation, Parapatellar, Transpatellar

## Abstract

**Background:**

This study evaluated the influence of meniscal allograft transplantation (MAT) on knee joint mechanics during normal walking using finite element (FE) analysis and biomechanical data.

**Methods:**

The study included 20 patients in a transpatellar group and 25 patients in a parapatellar group. Patients underwent magnetic resonance imaging (MRI) evaluation after lateral MAT as a baseline input for three-dimensional (3D) and FE analyses. Three different models were compared for lateral MAT: intact, transpatellar approach, and parapatellar approach. Analysis was performed using high kinematic displacement and rotation inputs based on the kinematics of natural knees. ISO standards were used for axial load and flexion. Maximum contact stress on the grafted menisci and maximum shear stress on the articular surface of the knee joint were evaluated with FE analysis.

**Results:**

Relatively high maximum contact stresses and maximum shear stresses were predicted in the medial meniscus and cartilage of the knee joint during the loading response for all three knee joint models. Maximum contact stress and maximum shear stress in the meniscus and cartilage increased on the lateral side after lateral MAT, especially during the first 20% of the stance phase of the gait cycle. The transpatellar approach was most similar to the intact knee model in terms of contact stresses of the lateral grafted and medial meniscus, as well as maximum shear stresses during the gait cycle. In addition, the transpatellar model had lower maximum contact stress on the menisci than did the parapatellar model, and it also had lower maximum shear stress on the tibial cartilage.

**Conclusions:**

Therefore, the transpatellar approach may reduce the overall risk of degenerative osteoarthritis (OA) after lateral MAT*.*

## Introduction

A meniscectomy may lead to early osteoarthritis (OA). Meniscus preserving techniques, such as repairs or partial resections, have become mainstream treatments for meniscus injuries. The clinical outcomes after subtotal or total meniscectomy are well known, and meniscal allograft transplantation (MAT) has been performed to prevent the development of arthritic degeneration. However, comparisons of published results remain troublesome because of the variety of associated procedures, allograft preservation methods, graft fixation techniques, clinical scoring systems, and durations of follow-up. Based on the available short- and medium-term data, it is generally accepted that MAT relieves pain and improves function in symptomatic meniscectomized knees [1–3].

The keyhole method with a parapatellar approach has been frequently used for lateral MAT. However, it is not always possible to insert meniscal allografts in an anatomically correct position when using the parapatellar approach [4, 5]. The transpatellar approach, which allows surgeons to achieve anatomical placement of the meniscal allograft, has recently been introduced to overcome the weaknesses of the parapatellar approach [6]. Some studies have indicated that the parapatellar approach is the best method for achieving correct anatomical positioning [4, 5]; however, there has been no biomechanical study comparing transpatellar and parapatellar approaches.

It is impractical to use experimental measurements to directly evaluate stress distribution in the lateral meniscus and tibial cartilage after lateral MAT. However, this limitation can be overcome by finite element (FE) analysis. Direct in vivo measurement of stress and strain at the knee cartilage is challenging. Therefore, the FE method has been used to determine stresses and strains within the knee joint [7–9]. Recent FE models of knee joints that had undergone meniscectomy only considered axial types of static loading conditions [10, 11]. The developed FE knee models provide significant insight into stress distribution, strain distribution, and contact kinematics at the knee joint. These models have been used to investigate the effect of ligament injury [12] and meniscectomy [11] on contact stress and strain at the knee joint. Models including gait cycle (walking) loading did not consider every rotation and translation movement of the knee joint. Saveh et al. developed a malalignment model that mimics normal walking [13]. This approach has also been employed by Mononen et al., who modeled the cartilage and meniscus with a partial meniscectomy [14]. Most studies using FE analysis have been limited to the cartilage and meniscus and have not studied ligaments. No study has employed real patient radiology in FE analyses, and no model has been developed to allow clinicians to perform virtual surgery. Kang et al. have referred to data from real patients, but static loading in full extension was applied rather than gait cycle loading [15]. To our knowledge, no previous studies have assessed the effect of anatomically correct positioning on degenerative OA in knee joints after MAT.

In the present study, an FE model of lateral MAT was developed to include bony structures (femur, tibia, and fibula) and ligaments (anterior and posterior cruciate ligaments, medial and lateral collateral ligaments). 3D in vivo analysis was performed using magnetic resonance imaging (MRI) to determine the correct position for the lateral meniscus, followed by lateral MAT without virtual surgery. Maximum contact stress on the grafted meniscus and stress exertion (maximum shear stress) on the tibial cartilage after lateral MAT were also investigated. The degenerative OA effects on grafted menisci could be evaluated by the maximum contact and shear stresses because these two parameters are closely associated with degenerative OA of the knee joint [16, 17]. Loading conditions included normal level walking for healthy humans and knee models of the transpatellar and parapatellar approaches. We hypothesized that accurate anatomical positioning achieved with this technique is closer to normal knee kinematics, leading to better functional outcomes in knee joints.

## Methods

### Three-dimensional analysis

After the hospital’s institutional review board authorized this study, patient data was used to develop transpatellar and parapatellar FE models. All patients underwent an MRI examination (Achieva 1.5 T; Philips Healthcare, Netherlands) on the operated knee joint at 2 years postoperatively. MRI scans were obtained in the sagittal plane at 0.4 mm slice thickness. For fat saturation, the MRI consisted of an axial proton density (PD) sequence. High-resolution settings were used for the spectral presaturation inversion recovery (SPIR) sequence (TE 25.0 ms, TR 3,590.8 ms, acquisition-matrix 512 × 512 pixels, NEX 2.0, field of view 140 × 140 mm). MRI images were used to reconstruct the tibia, menisci, ligaments, and bony bridge. All 3D reconstruction processes were performed manually with Mimics software (version 14.1; Materialise, Leuven, Belgium).

**Fig. 1:**
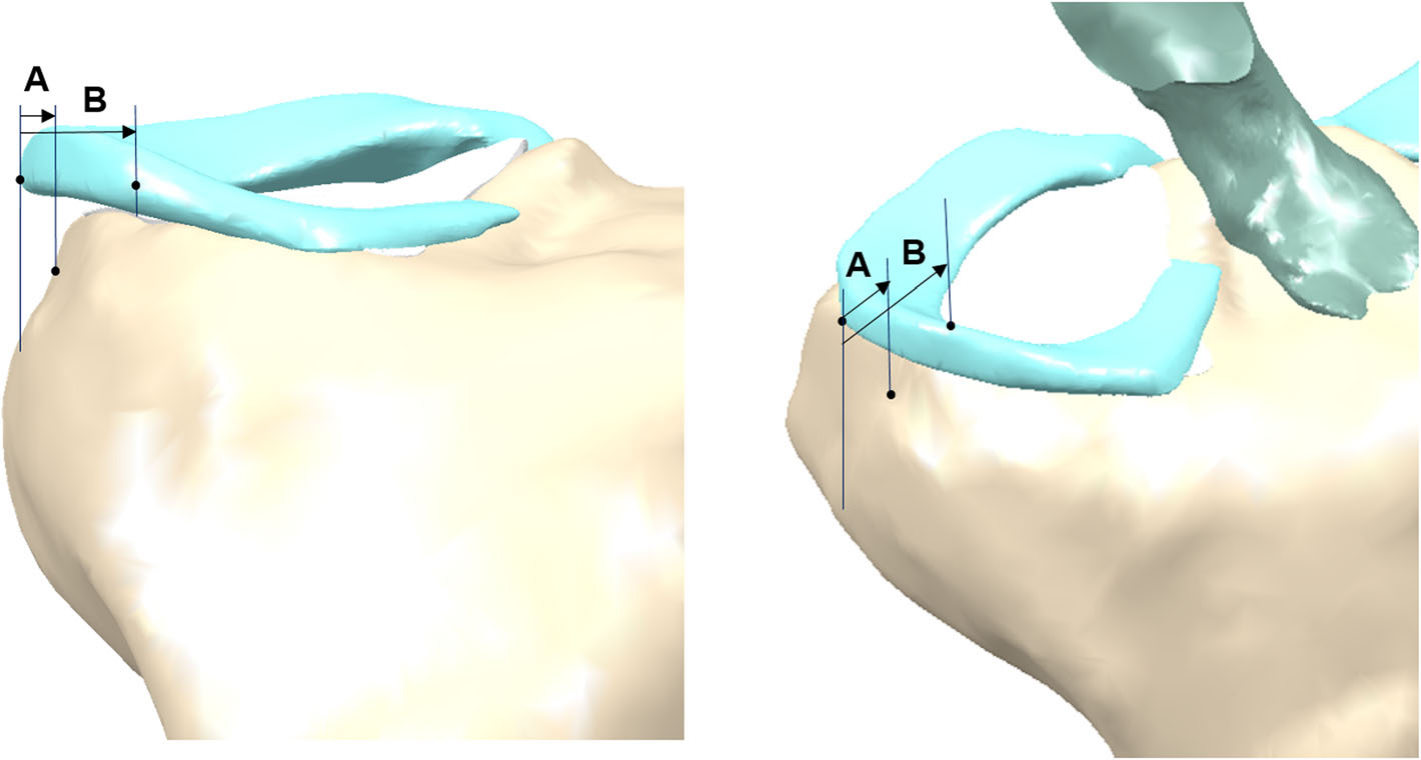
Measurement of the meniscal extrusion. Three-dimensional reconstruction image showing how extrusion was measured. A, width of the extruded meniscus; B, width of the entire meniscus; A/B, relative percentage of extrusion

**Fig. 2:**
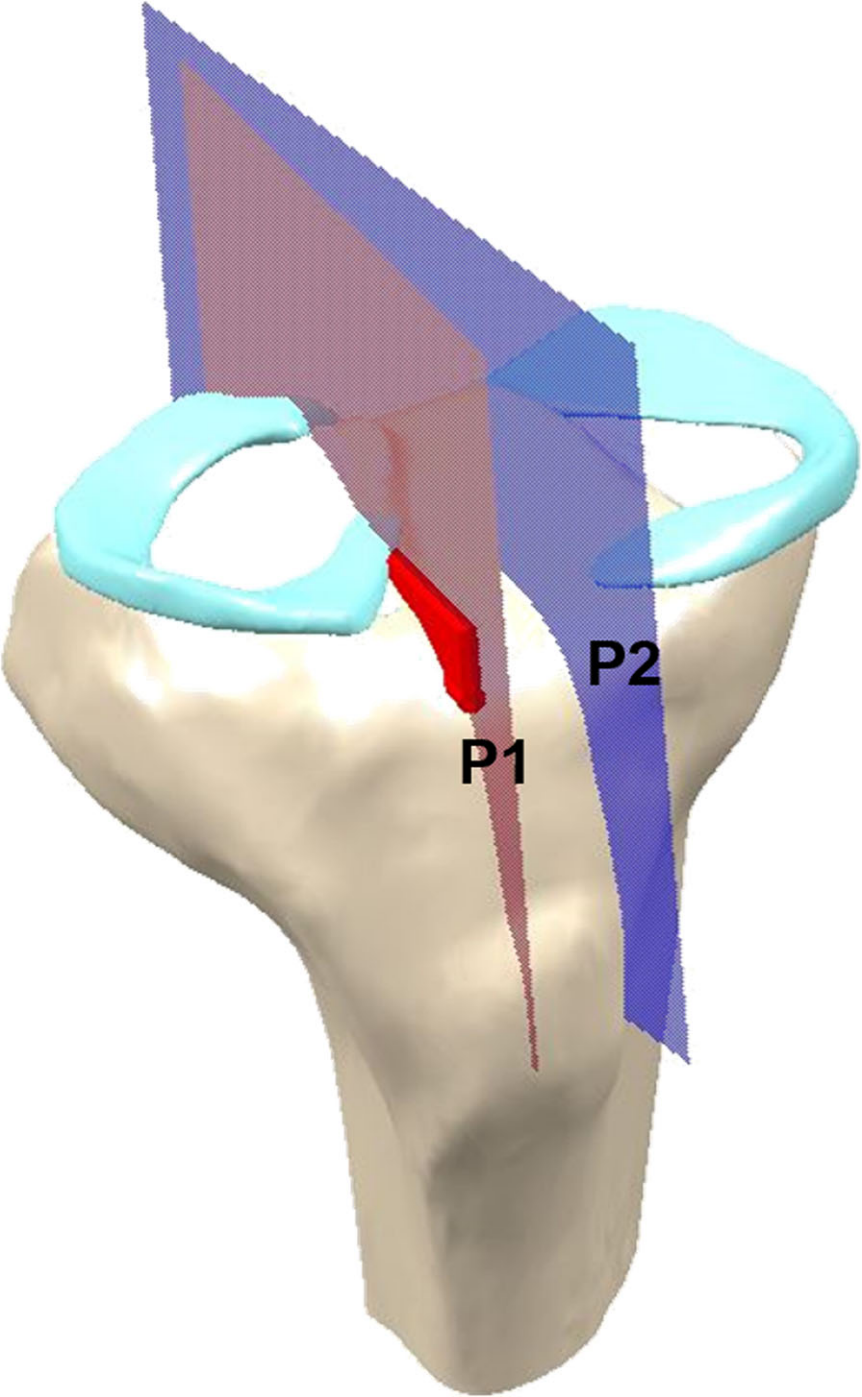
Parameters of the grafted meniscus. P1, plane of the bony bridge of meniscal allograft; P2, plane of the central line connecting each tibial attachment sites of the anterior and posterior cruciate ligaments. The obliquity of bony bridge and distance from the entry point of bony bridge to the center of the tibial plateau were determined by the angle and distance between P1 and P2

Extrusion of the mid-body of the meniscal allograft was measured on 3D images showing maximum extrusion. Extrusion was measured as the distance between the outer edge of the articular cartilage of the lateral tibial plateau and the outer edge of the meniscal allograft. Relative percentage of extrusion (RPE) was calculated as the percentage of the width of the extruded menisci compared to the entire meniscal width to provide a standardized measure for knees of different sizes (Fig. 1). To measure the parameters of grafted menisci, including obliquity of the bony bridge and distance from the entry point of the bony bridge to the center of the tibial plateau, two planes were obtained in the 3D reconstruction image: P1 was defined as the plane of the bony bridge of the meniscal allograft, and P2 was defined as the plane of the central line connecting the tibial attachment sites of the anterior and posterior cruciate ligaments (Fig. 2). The obliquity of the bony bridge was determined by the angle between P1 and P2, and the distance between P1 and P2 was used to determine the distance from the entry point of the bony bridge to the center of the tibial plateau.

### Finite element analysis

#### Intact model

**Fig. 3:**
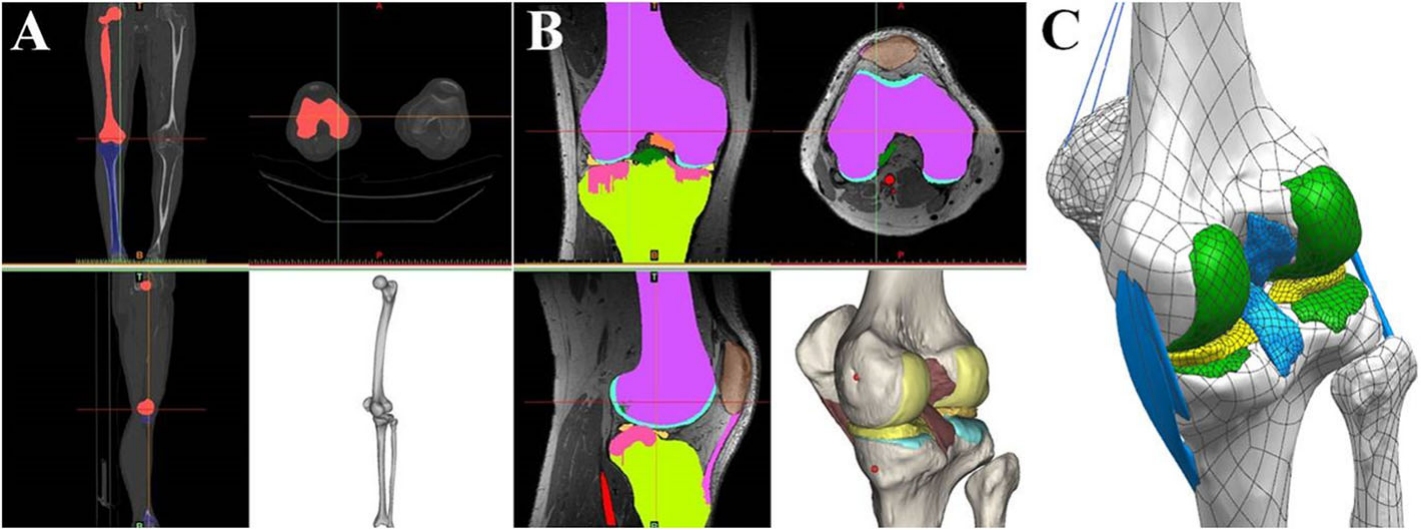
Methodology for the 3D modeling of the intact knee. **a** 3D bone reconstruction. **b** 3D soft tissue and ligament reconstruction. **c** 3D model modification

A 3D FE model of a healthy lower extremity was developed from computed tomography (CT) images obtained with a light speed volume CT scanner (VCT; GE Medical Systems, Milwaukee, WI, USA). CT scanning was performed with 0.1 mm slices from a 34-year-old male (height 178 cm, weight 75 kg). Digital CT data was imported into Mimics software (Materialise), which was used to generate 3D geometrical surfaces of the femur, tibia, fibula, and patella at full extension. The medial and lateral menisci, femoral cartilage, patellar tendon, and four major ligaments (anterior and posterior cruciate ligaments, medial and lateral collateral ligaments) were developed manually using 3D reconstruction models based on MRI. These images were used to reconstruct the femur with a distal thickness of 10.2 cm and the tibia with a proximal thickness of 7 cm. To match the positional coordinates of each model, anatomical reference points were defined as the central point of the femur diaphysis, midpoint of the trans-epicondylar axis, and intercondylar notch in the reconstructed CT and MRI models. The process of combining reconstructed CT and MRI models with positional alignment for each model was performed with Rapidform commercial software (version 2006; 3D Systems Korea, Inc., Seoul, South Korea). The initial graphics exchange specification (IGES) files exported from Mimics were entered into Unigraphics NX (version 7.0; Siemens PLM Software, Torrance, CA, USA) to form solid models for each femur, tibia, fibula, patella, and soft tissue segment. The solid model was then imported into Hypermesh (version 8.0; Altair Engineering, Inc., Troy, MI, USA) to generate an FE mesh. The FE mesh was analyzed with ABAQUS software (version 6.11; Simulia, Providence, RI, USA). Methodology for the 3D modeling of the intact knee is illustrated in Fig. 3.

Bones in this model were assumed to be rigid because the bone is stiffer than relevant soft tissues, and it had minimal influence in this study [7]. Therefore, each bony structure (femur, tibia, fibula, and patella) was represented by a primary node located at its center of rotation at full extension. FE models of soft tissue included the menisci, articular cartilage, patellar tendon, and four major ligaments. Articular cartilages were defined as isotropic, linear elastic materials with Young’s modulus of 15 MPa and Poisson’s ratio of 0.47 due to the time-independent and simple compressive load applied to the knee joint [18]. Menisci were modeled as transversely isotropic, linearly elastic, homogeneous material with Young’s modulus of 120 MPa in the circumferential direction and 20 MPa in the axial and radial directions. Poisson’s ratio was 0.2 in both circumferential and radial directions and 0.3 in the axial direction [19–21]. To simulate meniscal attachments, each meniscal horn was fixed to the bone using linear spring elements (element type = SPRINGA) with a total stiffness of 2000 N/mm at each horn [22, 23]. Interfaces between the cartilage and bones were modeled as fully bonded. Contact was modeled between the femoral cartilage and meniscus, meniscus and tibial cartilage, and femoral and tibial cartilage for both the medial and lateral sides, resulting in six contact pairs. A full large-strain formulation was considered with general contact conditions that included finite sliding. Kinematic constraints on the contact overclosure were approximated so that the nodes on the slave surface did not penetrate the master surface. The linear penalty method was used to determine the values of contact stress at each surface node. The coefficient of surface friction was 0.02, which is in the normal range for human articular joints [24]. The four major ligament models were defined as hyperelastic rubber-like materials, which represent nonlinear stress-strain relations [25, 26]. A hyperelastic model is generally used in engineering to represent large, incompressible deformation. The model is characterized by a strain energy potential function that is represented by equations [26]. The polynomial form of strain energy potential was chosen from the ABAQUS material library. Biological soft tissues are usually exposed to a distribution of in vivo residual stresses as a consequence of continuous growth, remodeling, damage, and viscoelastic strains. The initial ligament strain model was developed based on the results of a previous study [7].

#### Transpatellar and parapatellar models

**Fig. 4:**
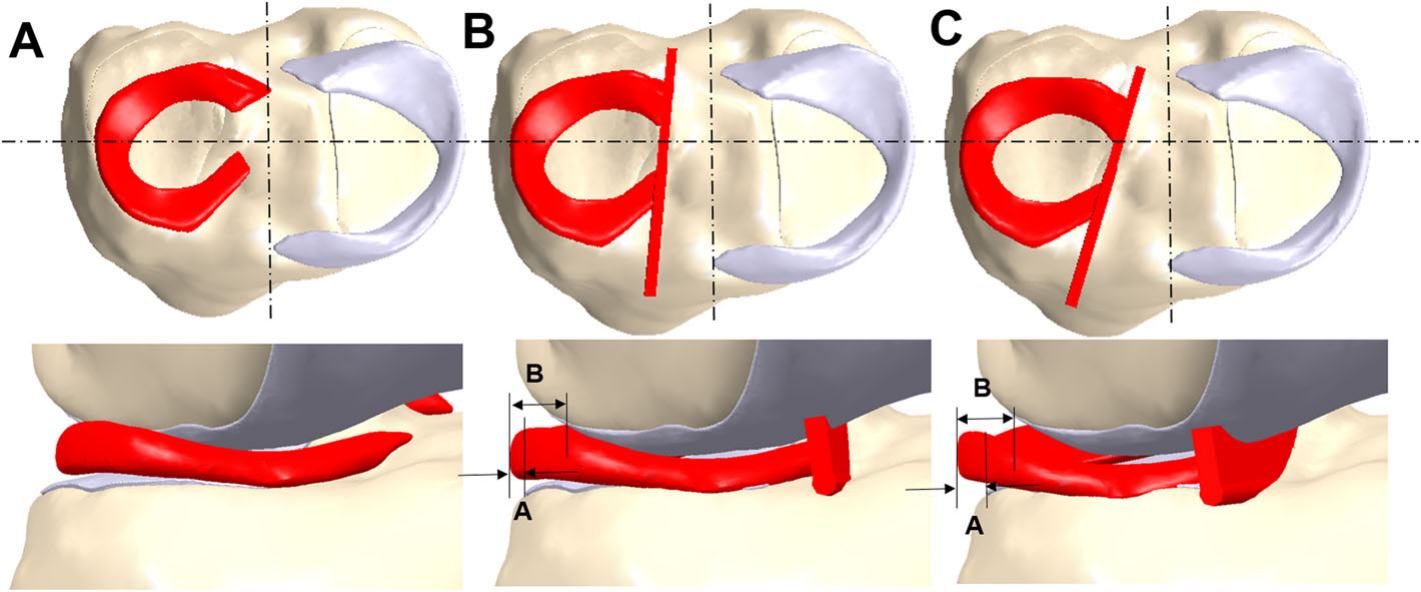
FE models used in analyses. **a** Intact model. **b** Transpatellar model. **c** Parapatellar model

For realistic simulation, average values from 45 patients (transpatellar, 20; parapatellar, 25) were obtained using 3D analysis for the RPE values, obliquities of the bony bridge, and distances from the entry point of the bony bridge to the center of the tibial plateau (Fig. 4). This geometric information was compared to the results from a previous paper (Table 1) [27]. An FE model that included realistic morphology was developed through this analytical process. Contact conditions for the transpatellar and parapatellar models were identical to those for the intact model. The medial meniscus, as in the intact model, was fixed to the bone using linear spring elements. However, the lateral meniscus was fully bonded to the bone using a bony bridge. To ensure complete simulation, mesh convergence tests were performed. Mesh convergence data was reported in our previous study [15, 28].

**Table 1:** Parameters of grafted meniscus

	Parapatellar group	Transpatellar group	*p* value
Width of entire meniscus (mm)	10.32 ± 1.23	10.68 ± 1.75	.343
Width of extruded meniscus (mm)	4.32 ± 0.58	3.00 ± 0.61	< .001
RPE (%)	42.48 ± 7.82	28.21 ± 4.49	< .001
Angle (°)	16.69 ± 2.68	5.29 ± 1.55	< .001
Distance (mm)	16.68 ± 2.56	10.81 ± 1.37	< .001

Values are shown as mean ± standard deviation. *RPE* relative percentage of extrusion, *Angle* angle between the bony bridge and center of tibial plateau, *Distance* distance from the entry point of bony bridge to the center of the tibial plateau

#### Loading and boundary conditions

The FE simulation included three types of loading conditions, corresponding to the loads utilized in the experiment for model validation and predictions of daily activity loading scenarios. Under the first loading condition, 150 N was applied to the tibia with 30° and 90° flexion of the FE knee joint to measure anterior-posterior (AP) tibial translations [29]. Additionally, a second axial loading of 1150 N was applied to the model to obtain the contact stresses and compare them to those reported in a published FE knee joint study [16].

**Fig. 5:**
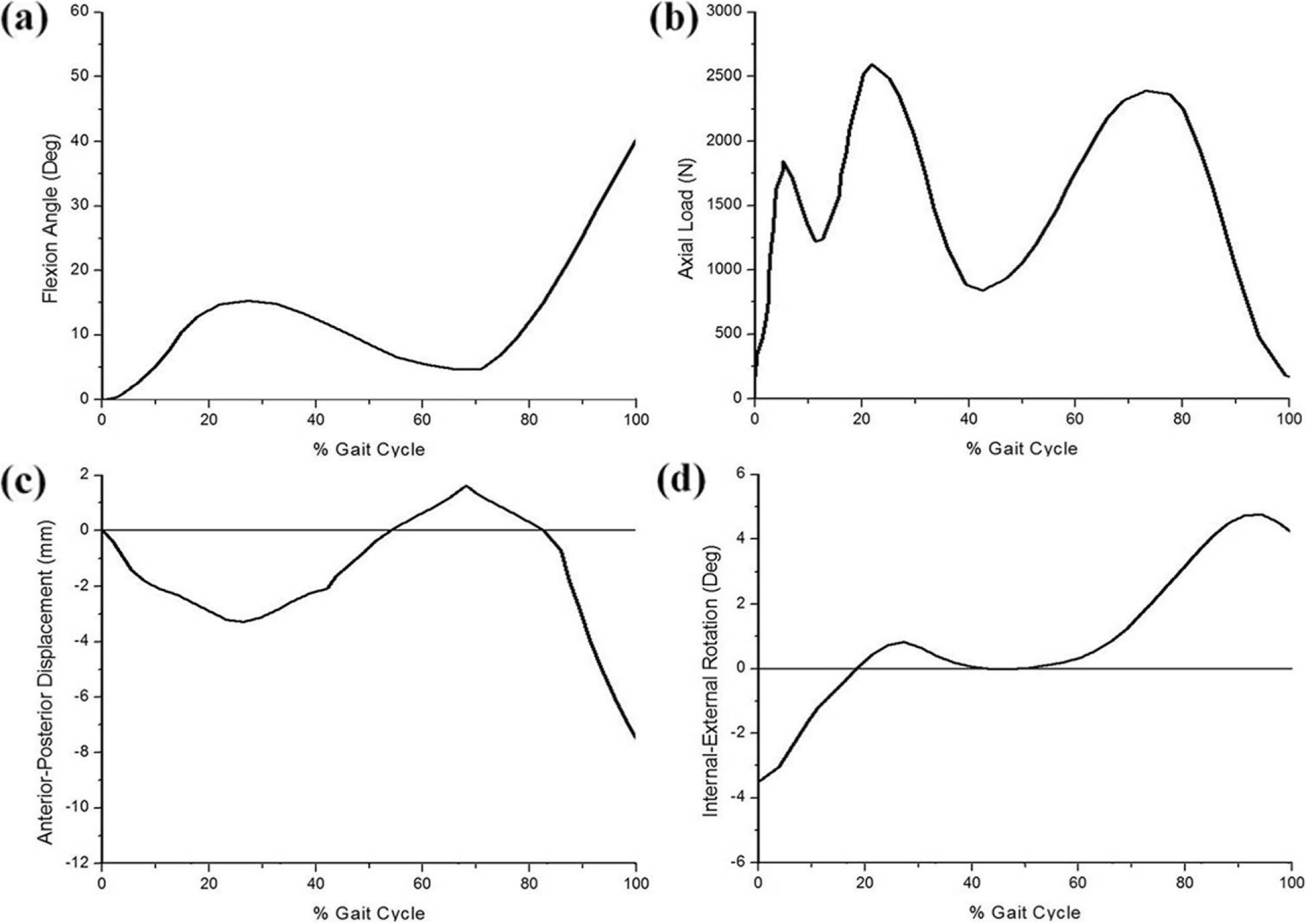
FE model inputs as a function of the gait cycle. **a** Flexion angle. **b** Axial load. **c** Anterior-posterior displacement. **d** Internal-external rotation

Third loading conditions were used to generate a model that can predict contact stress and shear stress in the knee joint during the instance phase of a loaded gait cycle (ISO 14,243) [30]. Femoral axial loading (maximum 2600 N) and extension-flexion (0°~58°) input profiles were adopted from the ISO 14,243 standard for all FE analysis studies (Fig. 5). Tibial rotation was displacement-controlled with an internal-external (IE) rotation of ± 5°, based on the natural knee kinematics described by Lafortune et al. [31] who collected data on healthy patients without replacement prostheses. Maximum contact stress was evaluated at the menisci. Maximum shear stresses were evaluated at the articular surface. Results for each of the three knee FE models (intact, transpatellar, and parapatellar) were compared.

## Results

### Validation

For validation, the natural FE model was compared to the experiments using its FE model subject. Under loading conditions with a flexion of 30°, the anterior tibial translation was 2.83 mm in the experiment and 2.54 mm in the FE model. The posterior tibial translation was 2.12 mm in the experiment and 2.18 mm in the FE model. With a flexion of 90°, the anterior tibial translation was 3.32 mm in the experiment and 3.09 mm in the FE model. The posterior tibial translation was 2.64 mm in the experiment and 2.71 mm in the FE model. It showed a good agreement between the experiments and the FE model [29]. In addition, the natural FE model was validated through a comparison with results from a previous computational study [16]. Average contact stresses of 3.1 and 1.53 MPa were found on the medial and lateral meniscus, respectively, under an axial load of 1150 N. Both values were within 6% of the 2.9 and 1.45 MPa contact stress values reported in [16]. These minor differences may be a result of geometrical variations between different studies, such as differences in the thickness of the cartilage and meniscus. The consistency between the validation results and results reported in the literature demonstrates the validity of the results obtained from the FE model utilized in this study.

### Comparison of maximum contact stress among models

The effects of the three FE models on maximum contact stress at the menisci were investigated during the stance phase of the gait cycle.

**Fig. 6:**
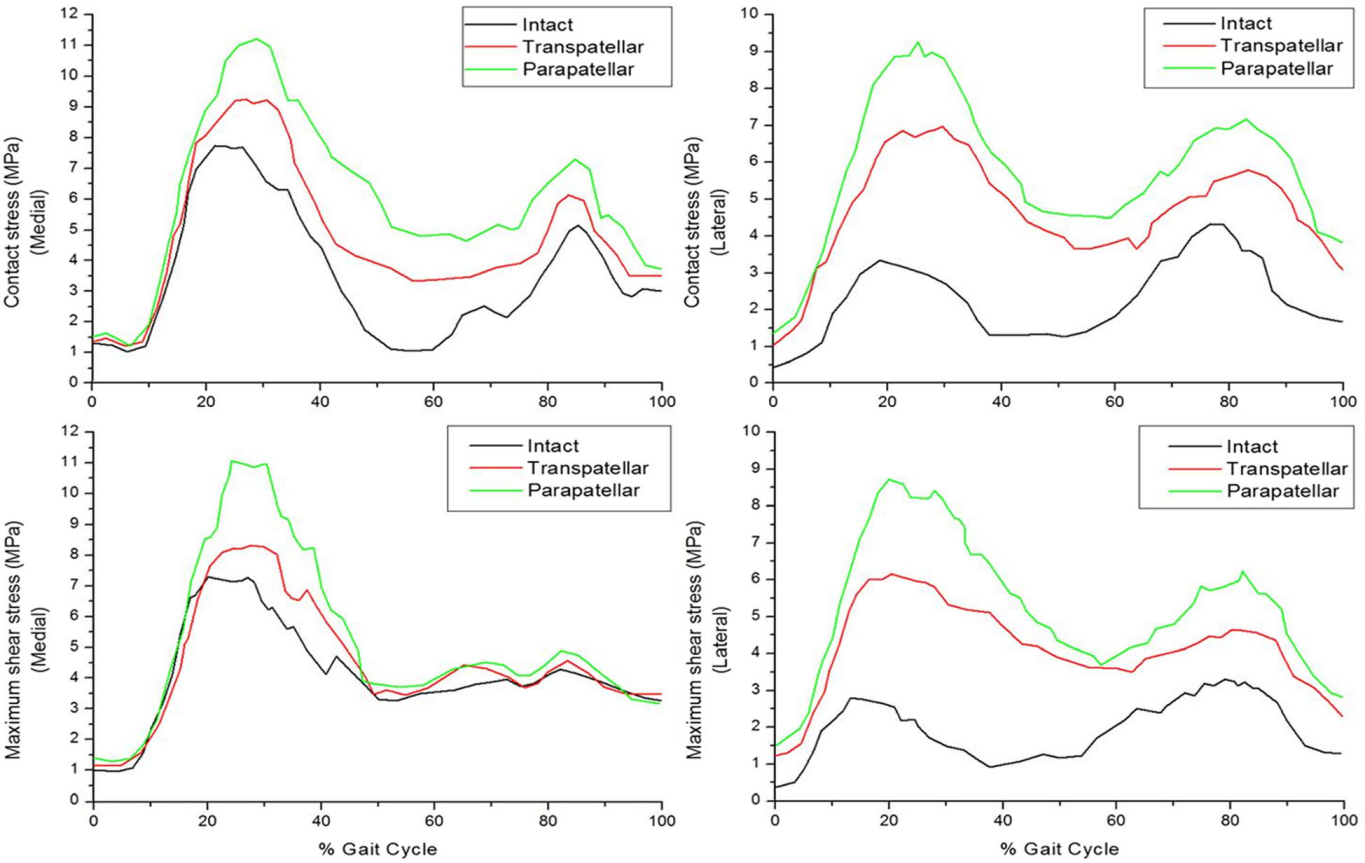
Comparison of maximum contact stress and maximum shear stress in the three models

In the intact model, contact stress values were significantly different on the medial and lateral sides of the menisci during the stance phase of gait (Fig. 6). The maximum contact stress of the medial meniscus was higher than that of the lateral meniscus. Under axial force, peak contact stress values of the three FE models in the medial compartment were found at around 20~30% of the stance phase of the gait cycle. Maximum contact stress on the lateral meniscus was 113.6% higher in the parapatellar model than in the intact model and 60.7% higher in the transpatellar model. The results indicate that the maximum contact stress on the medial meniscus also increased, in addition to that of the lateral compartment. The second maximum contact stress was partly transferred from the medial side to the lateral side of the meniscus in the intact model. Consequently, contact stress was similar for the lateral and medial sides. The second maximum contact stress transferred in the transpatellar and parapatellar models was lower than that of an intact knee. In particular, there is less load transfer in parapatellar model compared to transpatellar model. These results indicate that contact stress should be in an anatomically correct position for lateral MAT. In other words, anatomically mid-position influences to load transfer during the stance phase of the gait cycle.

### Comparison of maximum shear stress among models

Figure 6 shows maximum shear stress in the articular surface of the three models during the stance phase of the gait cycle. Maximum shear stress in the tibial cartilage was 7.27 MPa in the medial intact model, while that in the medial meniscus was higher than in the lateral meniscus. Maximum shear stress of the lateral cartilage for all cases usually occurred at around 15~25% of the stance phase of the gait cycle. The maximum shear stress in the lateral tibial cartilage was 164.2% higher in the parapatellar model than in the intact model and 86.4% higher in the transpatellar model (Fig. 6). The parapatellar model demonstrated significantly increased maximum shear stress of the articular surface relative to the intact model.

## Discussion

The most important finding of this study is that transpatellar approach may reduce the overall risk of degenerative osteoarthritis (OA) after lateral MAT. Because the transpatellar model had lower maximum contact stress on the menisci than did the parapatellar model, it also had lower maximum shear stress on the tibial cartilage.

The effect of lateral MAT on knee joint mechanics was evaluated using computational modeling by implementing realistic information about normal walking. A 3D nonlinear FE model of the knee joint that consisted of bony structures and ligaments was developed. The main objective was to compare maximum contact stress on the grafted menisci and maximum shear stress on the articular surface of the knee joint during the stance phase of the gait cycle using three different FE models. Prior studies were mostly related to meniscectomy, although MAT likely leads to better functional outcomes in general [10, 11, 16]. In addition, only one FE model has been developed to provide virtual surgery or patient-specific FE analysis. This study uses patient data of postoperative MAT position 2 years after surgery to develop a more realistic FE model. Kang and Chun primarily used data from real patients to develop an FE model with single axial static loading but did not observe clinical relevance under gait cycle conditions [15]. In addition, Kim et al. showed transpatellar model and parapatellar model at postoperative MAT position 2 years after surgery to develop a more realistic FE model [32]. However, the aforementioned studies have limitation in simple static loading condition.

Our goal was to demonstrate the importance of anatomically correct positioning by comparing results of the transpatellar and parapatellar approaches with normal healthy knees during stance phase gait cycle loading conditions.

Significant differences in maximum contact stress and maximum shear stress were predicted for the medial and lateral sides of a normal knee joint with the intact model. This is especially true during the loading response of the stance phase of the gait cycle, when the maximum contact stress on the medial side is very high. According to the results of Kwon et al. [28], maximum contact stress increases as it becomes an axial force, which is similar to the trend discovered in the present study. Maximum contact stress on the menisci was higher in the parapatellar model than in the transpatellar model. The contact parameter is closely associated with degenerative OA of the knee joint [33]. Therefore, OA is more likely after the parapatellar approach. Interestingly, this study determined that the maximum contact stress in the medial compartment increased after lateral MAT. This phenomenon may have occurred due to changes in load transfer caused by the position of the lateral meniscus, consistent with previous results [34]. Mononen et al. emphasized the importance of the lateral meniscus in load transfer because contact stress in the lateral tibial cartilage initially increases after total meniscectomy [14]. The role of the meniscus in distributing knee joint forces was most significant during the loading response of the gait cycle. This load transfer mechanism could not be found in the analysis under the static loading condition. To observe the load transfer mechanism after MAT, dynamic loading condition such as gait cycle is required, not a simple static loading condition. The results from the stance phase of gait cycle analysis have shown the load transfer on menisci followed by LMT. Previous studies have suggested that maximum shear stresses can damage cartilage [16, 17]. The results of the present study showed that the maximum value of shear stress of the lateral tibial cartilage occurred at around 15~25% of the stance phase of the gait cycle. The maximum shear stress of lateral tibial cartilage increased by 86.4% with the transpatellar approach, a smaller increase compared to that of the parapatellar approach. Interestingly, the increase in maximum shear stress with lateral MAT was much smaller than with meniscectomy [10, 16]. The results indicate that with MAT, the maximum contact stress of the articular surface decreased with increased contact surface. Therefore, we accepted our hypothesis that lateral MAT is less likely to result in progressive degenerative OA than meniscectomy. Thus, lateral MAT has a more positive effect on degenerative OA than meniscectomy [32]. Compared to other previous studies for lateral meniscectomy, after lateral MAT surgery, the maximum contact stress and its quantitative increase are significantly lower [32]. However, this result shows the importance of ensuring an anatomically correct position for lateral MAT.

The present study has some limitations. First, cortical and cancellous bones were not simulated in all models. However, this has a minimal influence because the bone is stiffer than soft tissue [7, 10, 16]. Second, only the intact model was validated. The lateral MAT model should be validated in the future to provide more quantitative results. Third, in lateral MAT in vivo, the stiffness and shape of menisci are not constant. Although we assumed specific shapes and material properties for the menisci, the purpose of this study is to demonstrate that the transpatellar approach provides more clinically correct positioning 2 years after surgery using 3D in vivo analysis and to evaluate the effect of this correct positioning on OA. The results from the present FE model demonstrated a similar trend to the clinical findings [35]. Accurate stress and strain evaluation is important when simulating specific functions of the knee joint. Unlike all previous computational models in static loading condition [7, 10–12, 16], the presented computational model considered all possible rotations and translations of knee joints during normal human walking.

## Conclusions

Our model provides important information about the differences between stresses at articular cartilages and menisci. This study is also an advanced investigation using FE analysis for lateral MAT surgery, including realistic loading conditions. As well, this study is the primary and more advanced study using FE analysis for lateral MAT surgery including realistic normal walking loading condition. Unlike other studies that used FE analysis, we used patient data to develop the FE models and ensure realistic simulation. Both maximum contact stress and maximum shear stress were lower in the transpatellar model than in the parapatellar model. We have found the significant effect of correct anatomical position for lateral meniscus using finite element analysis during the stance phase of the gait cycle. Accurate anatomical positioning was determined to be very important for positive surgical outcomes. Therefore, the transpatellar approach may reduce the overall risk of degenerative OA after lateral MAT. This study provides a useful stance phase of gait cycle data that observes the role of different biomechanical factors on the contact stress and shear stress at the knee joint.

## Data Availability

Not applicable

## Abbreviations

3D: Three-dimensional
AP: Anterior-posterior
CT: Computed tomography
FE: Finite element
IE: Internal-external
MAT: Meniscal allograft transplantation
MRI: Magnetic resonance imaging
OA: Osteoarthritis
PD: Proton density
RPE: Percentage of extrusion
SPIR: The spectral presaturation inversion recovery
